# Muscle Oxygenation During Exercise in Patients with Peripheral Artery Disease: A Systematic Review

**DOI:** 10.3390/app16094348

**Published:** 2026-04-29

**Authors:** Zahra Salamifar, Farahnaz Fallahtafti, Iraklis I. Pipinos, Cody P. Anderson, Song-Young Park, Sara A. Myers

**Affiliations:** 1 Department of Biomechanics, University of Nebraska at Omaha, Omaha, NE 68182, USA; 2 Department of Surgery, University of Nebraska Medical Center, Omaha, NE 68198, USA; 3 Department of Surgery and VA Research Service, VA Nebraska-Western Iowa Health Care System, Omaha, NE 68105, USA; 4 School of Health and Kinesiology, University of Nebraska at Omaha, Omaha, NE 68182, USA; 5 Department of Cellular and Integrative Physiology, University of Nebraska Medical Center, Omaha, NE 68198, USA

**Keywords:** claudication, ischemia, macrovascular disease, microvascular disease, near-infrared spectroscopy (NIRS)

## Abstract

Peripheral artery disease (PAD) involves atherosclerotic obstruction of the leg arteries and impairs function and structure of lower-limb tissues. Although traditionally regarded as a large-artery (macrovascular) disorder, PAD includes significant microvascular disease in the affected musculature, together leading to impaired leg perfusion. Monitoring muscle oxygenation during exercise provides an indirect index of limb perfusion and exercise capacity, and tracking its kinetics with near-infrared spectroscopy (NIRS; a portable, non-invasive technique measuring real-time tissue oxygen saturation) helps elucidate mechanisms underlying walking limitations in PAD. We systematically searched PubMed, Web of Science, and the Cochrane Library (1985–2025) for studies employing NIRS to monitor muscle oxygenation in PAD patients before, during, and after exercise. Of 628 articles screened, 11 met the inclusion criteria. NIRS demonstrated reliability and validity for monitoring muscle oxygenation in PAD. During walking, PAD patients showed a much steeper decline in muscle oxygenation and delayed recovery to baseline. Resting muscle oxygenation did not differ between patients with PAD and controls. These dynamics reveal the pathophysiological interplay in which proximal/macrovascular and distal/microvascular disease limit oxygen delivery and utilization in skeletal muscle. Accordingly, NIRS offers a sensitive, non-invasive tool to evaluate macro- and microvascular disease burden and monitor therapeutic response in PAD.

## 1. Introduction

Peripheral artery disease (PAD) is a cardiovascular condition that occurs when atherosclerosis narrows or obstructs the arteries supplying the lower extremities [1–4]. Muscle oxygenation is a valuable indicator of oxygen delivery and utilization, particularly in the calf muscles of patients with PAD, who experience reduced blood flow from large-artery blockages (macrovascular disease) as well as microvascular pathology and dysfunction [5–10]. PAD is a chronic disease in which oxygen supply to the leg tissues, especially during walking, cannot meet oxygen demand [11]. This persistent imbalance over months and years causes progressive structural, physiological, and functional injury to skeletal muscle, skin, adipose tissue, blood vessels, and nerves [11,12]. Together, this ischemic mismatch and tissue damage produce the hallmark manifestations of PAD, namely, intermittent claudication and chronic limb-threatening ischemia [13,14]. Claudication, the most common clinical symptom of PAD [15,16], occurs because patients cannot increase blood flow to skeletal muscle during walking, owing to macro- and microvascular disease [15,17]. This leads to muscle dysfunction, disabling leg pain, and severe walking impairment. Given that impaired perfusion underlies these symptoms and that skeletal muscle is the most active, oxygen-consuming tissue during walking, monitoring muscle oxygenation provides a practical and non-invasive way to assess both regional blood flow and balance between oxygen delivery and utilization [18–20]. During walking, rising metabolic demand in the working muscles pushes oxygen consumption beyond supply, so tissue oxygen saturation (StO_2_) falls (ischemia) [21]. Once activity stops, oxygen supply once again exceeds demand, and StO_2_ returns toward baseline (reperfusion). Calf muscle StO_2_ is influenced by several factors, including macro- and microvascular lesions, endothelial function, the efficiency of oxygen extraction by myofibers, and the efficiency of oxygen utilization by mitochondria [10].

Patients with PAD show altered physiological responses to physical activity [20,22,23], including reduced oxygen levels in the active skeletal muscles and delayed post-exercise calf muscle reoxygenation [24–28], with recovery rates slower than healthy individuals [29,30]. Monitoring dynamic changes in StO_2_ from rest to exercise and into recovery may therefore illuminate the pathophysiological basis of exercise limitation in patients with PAD [20]. Figure 1 illustrates a representative StO_2_ pattern in a patient with PAD during walking and recovery, highlighting commonly analyzed metrics.

Near-infrared spectroscopy (NIRS) is a well-validated, non-invasive method for measuring muscle (tissue) oxygenation, and it has been incorporated into wearable devices [32]. NIRS provides real-time insight into the balance between oxygen supply and demand in various conditions, including exercise [19,33,34], and low-flow states, including hypo-perfusion of organs and limbs due to different types of shock and blood flow limitations [33,35–38]. NIRS cannot differentiate between oxygen bound to myoglobin (Mb) and oxygen bound to hemoglobin (Hb) (Table 1). As a result, researchers report composite measures to represent overall tissue oxygenation [10,33,34,39]. Common NIRS-derived variables include StO_2_ (tissue oxygen saturation), SmO_2_ (muscle oxygen saturation), tissue oxygenation index (TOI), and tissue saturation index (TSI) (Table 1) [34,40–42]. In this manuscript, we use StO_2_, oxyhemoglobin (O_2_Hb), and deoxyhemoglobin (HHb) as primary indicators of muscle oxygenation, representing the combined oxygen content of Hb in blood and Mb in muscle cells [10,33,34,39]. Although this aggregate view cannot separate the individual contributions of oxygen bound to Hb versus Mb, it remains essential for monitoring tissue oxygen levels during exercise.

NIRS has been used to monitor muscle oxygenation at rest, during walking, and in recovery [29,46–52]. Monitoring muscle oxygenation changes with NIRS can help clinicians and researchers assess the effects of exercise in both healthy and clinical populations [51–57]. Studies have examined a range of skeletal muscles to reveal physiological adjustments during activity [47,58–65]. A few studies have investigated muscle oxygenation responses to high-intensity or resistance exercise training, showing variations in oxidative responses across different muscle groups [47–49,66]. Age-related comparisons indicate slower recovery in older adults [67–69]. Finally, marked alterations in muscle oxygenation have been observed in patients with PAD, before, during, and after exercise [20,70]. This review summarizes the effects of exercise on muscle oxygenation across different muscle groups, identifies gaps in current knowledge, and outlines future applications of muscle oxygenation monitoring in patients with PAD.

## 2. Materials and Methods

Preferred Reporting Items for Systematic Reviews and Meta-Analysis (PRISMA) is a guideline designed to ensure transparency, completeness, and accuracy in systematic reviews [45]. According to the PRISMA checklist, we conducted a systematic review of studies reporting StO_2_ during exercise in patients with PAD. The research question was formulated according to the Population, Intervention, Comparison, and Outcomes (PICO) strategy (Table 2) [71,72]. The review protocol was registered in the PROSPERO database (ID: CRD420251061973).

### Search Strategy

Relevant articles between 1 January 1985 and 20 March 2025 were searched using the PubMed, Web of Science, and Cochrane Library databases. The MeSH terms included “Infrared Spectroscopy,” “Muscle Oxygenation,” “Perfusion,” “Peripheral Artery Disease,” “Exercise Therapy,” “Gait Analysis,” “Intermittent Claudication,” “Lower Extremity Artery Disease,” “Supervised Exercise Therapy,” “Vascular Disease,” “Oxygen Consumption,” “Non-invasive Measurements,” “Muscle Oxidative Metabolism,” and “Exercise Physiology”.

### Inclusion and exclusion

Manuscripts were eligible for inclusion if they: (1) used NIRS to monitor muscle oxygenation during exercise; (2) included comparisons of patients with PAD and non-PAD controls; (3) evaluated at least three claudicating patients; (4) were written in English; (5) studied human subjects; and (6) included participants aged 60 years or older. Exclusions were based on screening the abstract and then the full manuscript to confirm lack of relevance. Articles that used magnetic resonance, ultrasound, and biopsy to assess muscle oxygenation without also using NIRS were excluded.

### Primary and secondary outcomes

The primary outcome of this manuscript was muscle oxygenation in patients with PAD compared with healthy age-matched individuals, as measured by NIRS before, during, and/or after exercise. The secondary outcome was the validity and reliability of NIRS for measuring muscle oxygenation.

### Quality control

Each article’s methodological quality was examined using the “Critical review form: quantitative studies” [73–75].

## 3. Results

Keyword searches identified 628 potential articles in the selected databases (Figure 2). After screening the titles, abstracts, and full contents, 11 articles met the inclusion criteria. No additional articles were identified through reference list searches.

### Descriptive Analysis

Table 3 summarizes the detailed methodological quality of the included studies. All 11 studies clearly stated the purpose, used the relevant literature, justified the sample size, reported the statistical significance, conducted an appropriate analysis, and reported the conclusion based on methods and results. However, the sample size justification was not reported in nine studies. Additionally, the number of dropouts was not reported in five studies [9,46,76–78].

Table 4 provides detailed information on participant numbers, study objectives, targeted muscles, measured parameters, and primary outcomes. Among the included studies, the most common study design was observational (n = 4) [9,32,46,80]. The remaining seven studies used prospective comparative (n = 1) [81], cross-sectional (n = 1) [77], comparative (n = 1) [83], case–control (n = 1) [82], and controlled experimental (n = 1) [79] study designs. In total, 317 patients with PAD and 196 healthy controls were included. Treadmill walking was the exercise modality in seven studies [9,32,46,76–78,81]. One study used both plantar flexion and treadmill protocols [83]; two studies focused on plantar flexion exercise [79,82]; and one study used a toe flexion protocol [80]. In all the studies, NIRS was used during exercise to measure muscle oxygenation in one or more lower-extremity muscles. Compared with healthy controls, patients with PAD consistently demonstrated prolonged recovery times and greater variability in muscle oxygenation during post-exercise recovery [9,32,46,77–80,82,83].

NIRS was consistently identified as a valid and reliable technique for assessing muscle oxygenation during exercise. Validity outcomes included correlations with ankle-brachial index (ABI), ankle pressure, dorsalis pedis pressure, disease severity classification, and receiver operating characteristic analyses [80,81]. One study further assessed validity by comparing NIRS-derived recovery indices with 31P magnetic resonance spectroscopy recovery rates, demonstrating close agreement between the two methods [82]. Reliability was assessed using intra-class correlation coefficients, according to repeated measurement consistency during exercise, or by repeating the exercise recovery measurement using NIRS and ABI, as well as an intrasubject coefficient of variation, which demonstrated consistency in oxygen recovery between trials [78,80,82,83].

The calf muscle (gastrocnemius) was the most frequently monitored site (n = 9) [9,32,46,76–79,81,82]. One study assessed the lateral soleus during plantar flexion exercise, and the medial gastrocnemius during progressive treadmill testing [83], and another placed the NIRS sensor on the dorsum of the subjects’ feet [80].

There was a variation in the timing of muscle oxygenation recording across studies. Comerota et al. recorded oxygenation for 5 min before exercise, throughout treadmill walking, and for 20 min during recovery [77]. Fuglestad et al. measured oxygenation for at least 3 min before exercise, during graded treadmill testing, and for 30 min post-exercise [32]. McCully et al. measured oxygenation during plantar flexion exercise and for 3–6 min afterward; however, pre-exercise recording duration and recovery timing after treadmill testing were not reported [83].

## 4. Discussion

This study systematically reviews the effects of exercise on lower-limb muscle oxygenation in patients with PAD, summarizing findings before, during, and after exercise. To our knowledge, this is the first systematic review to examine muscle oxygenation across all three phases of exercise. The results demonstrated that resting muscle oxygenation was similar between patients with PAD and healthy controls [9,32]. In contrast, patients with PAD showed a steeper, more rapid, and more pronounced decline in muscle oxygenation during walking, followed by markedly prolonged recovery to baseline, indicating substantial hypoxia in PAD muscles during ambulation [9,32,46,77–83]. Fuglestad et al. further reported that patients with PAD experienced a decline in StO_2_ after an average of only 12 steps (range of 1–55 steps), indicating that calf muscle ischemia in patients with PAD occurs well before the onset of claudication symptoms and at unexpectedly short walking distances [32]. Park and colleagues also found greater StO_2_ reduction after treadmill walking in patients with PAD compared with age-matched healthy individuals but a smaller difference in the minimum StO_2_ [9]. These findings suggest that the exercise-evoked change in StO_2_, rather than resting values or the nadir alone, may be the most sensitive and physiologically relevant biomarker of PAD-related muscle hypoxemia [20,77].

Fuglestad et al. proposed that the markedly altered StO_2_ profiles and severe walking limitation in patients with claudication reflect the multiple interrelated pathological features of PAD [32]. The disease begins with the atherosclerotic narrowing of the large arteries (macrovascular disease), which restricts blood flow to the limbs [32]. In addition, patients exhibit microvascular abnormalities, including capillary basement membrane thickening, perivascular fibrosis, and impaired endothelial reactivity, which further limit oxygen and nutrient delivery [8,32,84–86]. Compounding these defects, the PAD muscle develops chronic ischemic myopathy marked by abnormal myofiber morphology, cytoskeletal disruption, mitochondrial dysfunction, oxidative damage, and fibrosis [14,87–94]. Together, these macrovascular, microvascular, and myopathic abnormalities converge to produce the characteristic StO_2_ abnormalities and exercise intolerance of claudicating patients [95].

Several factors influence StO_2_ dynamics during walking, including physical activity history and disease severity. Patients with a sedentary lifestyle showed faster time to reach the minimum StO_2_ during treadmill walking, compared with patients with a light-to-moderate exercise history [96]. Intra-patient comparisons show higher muscle deoxygenation rates in PAD-affected versus unaffected legs [32,97]. Furthermore, those with severe PAD (ankle-brachial index < 0.50 and Fontaine classification III or IV) showed greater StO_2_ reduction during walking [46], suggesting that the magnitude of exercise-induced StO_2_ decline correlates with disease severity [32,98].

Evidence from plantar and toe flexion exercise further supports these findings. Kemp et al. reported greater reductions in muscle oxygenation during plantar flexion at both 50% and 75% of maximal voluntary contraction, along with slower recovery in patients with PAD compared with controls [82]. Recovery-phase muscle oxygenation findings were consistent with previous reports [23,83,99]. Additionally, McCully et al. reported a significantly prolonged recovery time constant (reflecting slower oxygen resaturation) following plantar flexion exercise in the PAD limb compared with the legs of both young and older controls, indicating impaired oxygen delivery and reduced microvascular perfusion in patients with PAD [83]. In contrast, recovery time constants did not differ between healthy young and older adults, indicating fast recovery after plantar flexion exercise in healthy populations [83]. Similarly, Manfredini et al. reported significantly greater StO_2_ reductions during toe flexion exercise, quantified by the area under the oxygenation curve, in patients with PAD compared with healthy controls [80]. They attributed this finding to insufficient blood flow and oxygen delivery during toe flexion exercise [80]. Evidence related to toe and plantar flexion exercise remains limited, highlighting the need for further studies to clarify physiological responses and potential rehabilitation implications in PAD.

In patients with claudication, recovery of StO_2_ following treadmill walking exercises is slower than in healthy individuals, and this delayed recovery likely reflects impaired microvascular oxygen delivery capacity [9,32,46,76–78,83]. Park and colleagues suggested that microcirculatory dysfunction, characterized by reduced capillary recruitment, impaired endothelial-dependent vasodilation, and abnormal regulation of arteriolar tone, may limit the rapid restoration of oxygen supply once exercise ceases [9]. Although oxygen delivery through large vessels may be adequate at rest, the regulation of perfusion at the microvascular level is compromised [9]. This imbalance between oxygen resupply and muscle metabolic demand during recovery prolongs the StO_2_ recovery slope, underscoring the contribution of microvascular pathology to exercise intolerance in PAD [9].

Regarding measurement accuracy, NIRS has been recognized as a reliable and valid tool for monitoring peripheral circulation during and after exercise [28,32,42,46,80,81]. NIRS is also a well-established research tool for monitoring changes in blood flow and muscle oxygenation and evaluating the effectiveness of exercise [28,32,42,46,80,81]. Fuglestad et al. reported the ability of NIRS to generate similar calf StO_2_ measurements compared with other costly devices, supporting the feasibility of non-invasive measurement to assess muscle oxygenation [32]. Comerota et al. further reported a high correlation between recovery time, as measured by the ABI and NIRS, after exercise, indicating the effectiveness of NIRS in evaluating oxygen demand and delivery in muscles following exercise [77]. However, according to Kooijman et al., the recovery time measured by the ABI was faster than with NIRS. This difference arises because ABI recovery depends solely on blood flow restoration, whereas NIRS captures both blood flow and muscle metabolic recovery [78]. This distinction highlights an important advantage of NIRS over the ABI, as it provides a more comprehensive assessment of muscle recovery dynamics [78].

One particularly important aspect of the findings of NIRS studies is that although patients with PAD experience a much greater and earlier drop in StO_2_ during exercise, which strongly correlates with walking impairment, resting muscle StO_2_ (before exercise) does not differ significantly between patients with PAD and healthy subjects [32,79,100–102]. This finding is corroborated by several other methodologies. Specifically, contrast-enhanced ultrasound (CEU) demonstrates that resting microvascular blood volume and flow velocity are comparable between groups, but the ability to augment perfusion during exercise is blunted in PAD, reflecting impaired functional hyperemia [103–106]. Phosphorus magnetic resonance spectroscopy and BOLD MRI also confirm that resting muscle oxygenation and phosphocreatine recovery are similar, yet after exercise, PAD patients show slower phosphocreatine recovery and more pronounced hypoxia-triggered vasodilation, indicating reduced mitochondrial oxidative capacity and oxygen delivery under stress [107–109]. Collectively, these modalities reveal that the pathophysiological hallmark of PAD and claudication is an inability to increase muscle blood flow and oxygenation during exercise, while resting values remain largely preserved.

NIRS complements these modalities by providing a non-invasive, portable, and relatively inexpensive method for the continuous, real-time monitoring of superficial muscle oxygenation during rest, exercise, and recovery, without the need for contrast agents, radiation, or ultra-expensive imaging equipment [32,40,110,111]. NIRS is less operator-dependent than Doppler and ultrasound and is particularly well suited for repeated or longitudinal measurements, making it practical in both clinical and research settings [111,112]. However, its limitations include restriction to superficial muscle layers, sensitivity to adipose tissue thickness, an inability to directly quantify absolute blood flow, and lower spatial resolution than that of contrast-enhanced ultrasound or MRI [40]. By comparison, CEU provides higher spatial resolution and dynamic perfusion assessment, but it is operator-dependent and limited to superficial muscle groups and requires intravenous contrast, which may not be suitable for all patients, and a larger team to perform the test [113,114]. CEU image quality can be degraded by patient body habitus or motion, and quantitative perfusion parameters are not yet standardized across centers [115]. MRI enables comprehensive anatomical and functional evaluation of deeper muscle perfusion and microvascular flow, but it is expensive, time-consuming, less accessible, and sensitive to motion artifacts [116,117]. MRI may be contraindicated in patients with certain implants or severe renal dysfunction, and quantitative perfusion assessment is not routinely performed in clinical practice [117,118]. Doppler ultrasound (duplex) is widely available and reliable for macrovascular flow assessment, but it is highly operator-dependent and less accurate in the presence of heavy arterial calcification or multiple sequential lesions and cannot directly quantify microvascular or tissue-level perfusion [119,120]. In summary, NIRS is best positioned as a practical tool for non-invasive, dynamic assessment of superficial muscle oxygenation over long periods of time, while CEU, MRI, and Doppler ultrasound provide more detailed anatomic and perfusion data at substantially greater cost and complexity [112,121,122].

### 4.1. Methodological Heterogeneity Across Included Studies

An important consideration when interpreting these findings is the methodological heterogeneity across the 11 included studies. Exercise protocols varied widely: seven studies employed treadmill walking with differing speeds (1.5 to 3.2 km/h), inclines (0% to 10 degrees), and durations (symptom-limited vs. fixed time) [9,32,46,76,77,81,83], while the remaining used plantar flexion at 50–75% of maximal voluntary contraction [50,79,83] or toe flexion with progressive loading [80]. These protocol differences directly influence StO_2_ kinetics and complicate cross-study comparisons [123,124]. The NIRS devices also differed, ranging from research-grade continuous-wave systems (e.g., NIRO, RunMan, and PortaMon) to low-cost wearable sensors (Moxy), each with distinct algorithms and interoptode distances that affect tissue sampling depth. Outcome variables were inconsistent, with some studies reporting StO_2_ and others measuring O_2_Hb, HHb, tHb, or derived indices (TOI, TSI, and area under the curve), limiting the quantitative pooling of results.

Patient populations also differed in disease severity (Fontaine stages II–IV), comorbidity profiles, and sample sizes (7 to 80 PAD patients per study). The anatomical site of NIRS measurement varied, with most studies monitoring the gastrocnemius but others targeting the soleus or foot dorsum—sites with distinct vascular supply and adipose tissue thickness. Despite this heterogeneity, the consistent finding across all studies that PAD patients exhibit greater exercise-induced deoxygenation and prolonged recovery strengthens the overall conclusion. Future studies should adopt standardized protocols, devices, and outcome reporting frameworks to enhance comparability.

### 4.2. Clinical Translation of NIRS Findings

While NIRS convincingly detects macro- and microvascular dysfunction during exercise in PAD [9,32,112], clinical translation requires further consideration. The ankle-brachial index (ABI), though widely used, has well-documented limitations: it can be falsely elevated with medial arterial calcification (common in diabetes and chronic kidney disease), provides no microvascular information, and does not assess dynamic exercise-induced ischemia [125–127]. NIRS overcomes these limitations by directly measuring tissue-level oxygenation during exercise. Several clinical applications emerge from this review: NIRS could serve as a point-of-care screening tool when combined with simple exercise protocols; NIRS parameters could objectively quantify disease severity given the observed correlations with the ABI and walking capacity; NIRS monitoring could guide individualized exercise prescription by identifying critical ischemic thresholds; and serial NIRS measurements could monitor therapeutic response following revascularization or rehabilitation programs.

However, barriers remain before NIRS can be integrated into clinical workflows. There is no consensus on standardized measurement protocols, normative reference values, or clinically meaningful thresholds. Adipose tissue thickness affects signal quality, and NIRS is restricted to superficial muscle layers [112,128–130]. Multicenter validation studies with standardized protocols and prospective outcome data are needed to establish the diagnostic accuracy, prognostic value, and cost-effectiveness of NIRS-based assessment in PAD.

Study limitations included the inconsistent timing of baseline and recovery measurements, with only three studies reporting exact measurement intervals [32,77,83]. Comerota et al. measured muscle oxygenation for five minutes before and 20 min after exercise [73], while Fuglestad et al. reported three minutes of baseline recording and 30 min of post-exercise monitoring [32,97]. McCully et al. reported post-exercise measurements of 3–6 min but did not specify baseline recording duration [83]. Standardization of measurement protocols would improve comparability across studies. Furthermore, most included studies were observational, limiting the ability to assess causal effects. Future intervention-based and longitudinal studies are needed to better define the effects of specific exercise modalities and the long-term impact of exercise on muscle oxygenation in PAD.

## 5. Conclusions

In conclusion, this systematic review of 11 studies supports the use of NIRS-derived muscle oxygenation parameters during exercise as a sensitive and physiologically informative tool for characterizing PAD pathophysiology. Although resting muscle oxygenation does not differ between patients with PAD and healthy individuals, the dynamic response to exercise differs markedly. Patients with PAD consistently exhibit a faster and steeper decline in muscle oxygenation during exercise, followed by substantially delayed recovery. These exercise-induced oxygenation abnormalities reflect the convergence of macrovascular disease (atherosclerotic obstruction limiting bulk blood flow), microvascular dysfunction (impaired capillary recruitment, endothelial dysfunction, and perivascular fibrosis), and ischemic myopathy (mitochondrial dysfunction, oxidative damage, and muscle fiber degeneration), which together constitute the pathophysiological substrate of claudication.

From a clinical perspective, NIRS offers a non-invasive, portable, and relatively inexpensive method for assessing PAD at the tissue level, complementing ABI and imaging-based approaches. The observed relationship between NIRS-derived parameters and walking capacity suggests that these measures may serve as meaningful biomarkers of disease severity and functional limitation. In addition, the ability of NIRS to detect muscle deoxygenation early during exercise highlights its potential value for monitoring disease progression and therapeutic response.

These findings should be considered in the context of some limitations. The included studies were heterogeneous with respect to exercise protocols, NIRS devices, outcome measures, and patient populations, and most were observational with relatively small sample sizes. Standardized measurement protocols and larger prospective studies are needed to define normative values, establish clinically meaningful thresholds, and determine responsiveness to interventions such as revascularization, structured exercise therapy, and pharmacotherapy. Overall, NIRS appears well positioned as a practical and informative tool for assessing exercise-induced muscle oxygenation changes and for advancing the evaluation of disease severity and therapeutic response in PAD.

## Figures and Tables

**Figure 1.**
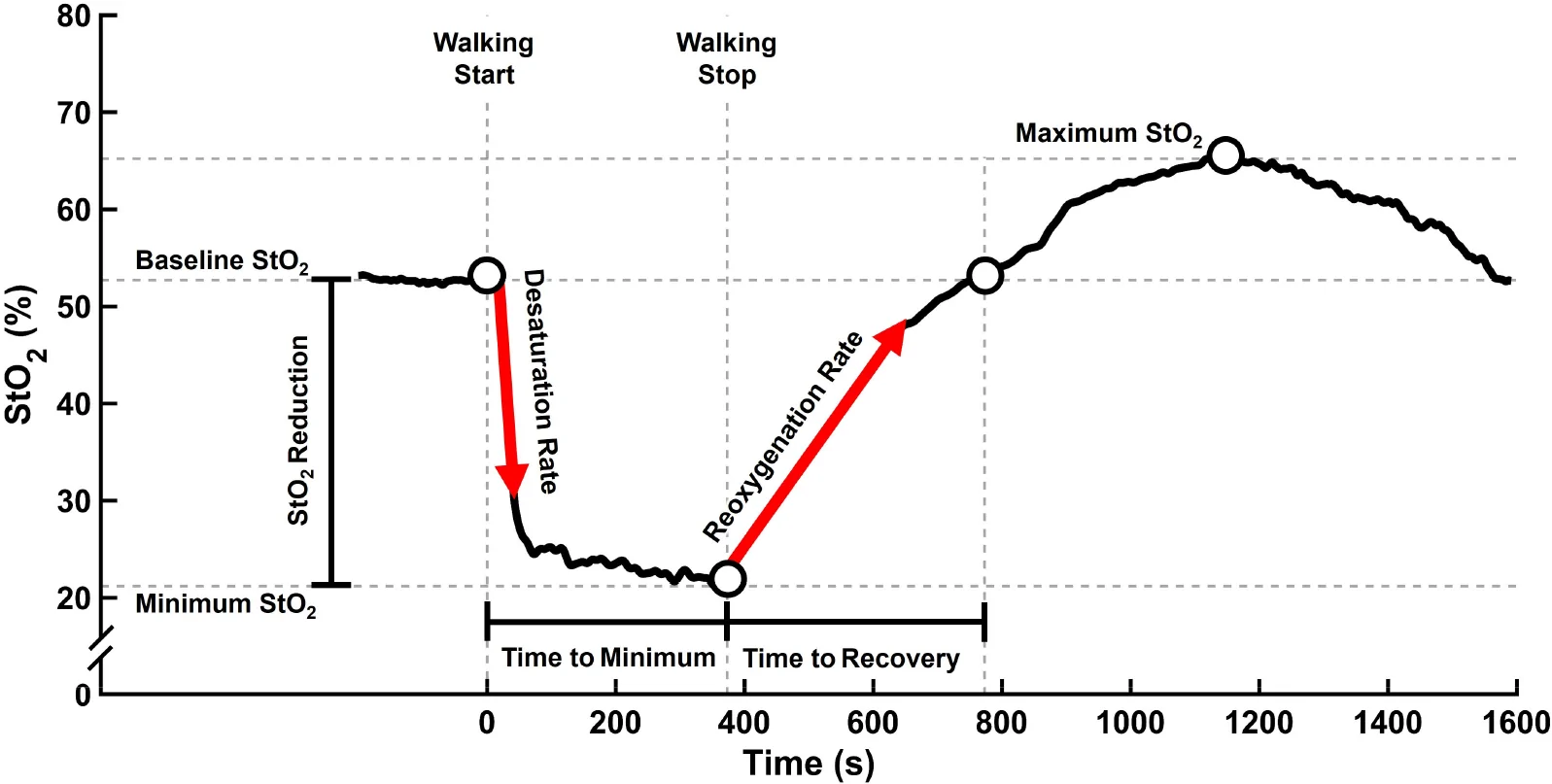
Representative StO_2_ trace for a patient with claudication due to peripheral artery disease during walking and common outcome measures. StO_2_ is the percentage of hemoglobin/myoglobin in the near-infrared spectroscopy sampling volume that is oxygenated. An StO_2_ of 100% indicates that all hemoglobin/myoglobin in the sampling volume is oxygenated, and an StO_2_ of 0% indicates that no hemoglobin/myoglobin in the sampling volume is oxygenated [31]. However, when using continuous-wave near-infrared spectroscopy systems, absolute StO_2_ values should be interpreted with discretion due to assumed scattering coefficients. Therefore, it cannot be assumed that full oxygen desaturation during walking occurs at an StO_2_ of 0% for all patients. In experiments in patients with peripheral artery disease, the near-infrared spectroscopy sensor is commonly adhered to the skin superficial to the medial gastrocnemius muscle. Prior to walking, the baseline StO_2_ is assessed as the steady-state StO_2_ in the standing position. Muscle oxygen desaturation during walking is captured with several outcome variables, such as StO_2_ reduction, desaturation rate, and time to minimum StO_2_. StO_2_ reduction is the difference between the baseline StO_2_ and the minimum StO_2_ achieved during walking. The desaturation rate is quantified as the slope of the linear portion of StO_2_ descent at the beginning of walking. Finally, time to minimum StO_2_ is the time elapsed from the onset of walking to when the minimum StO_2_ is achieved. StO_2_ recovery after walking is captured with several outcome measures, such as reoxygenation rate, time to StO_2_ recovery, and maximum StO_2_. The reoxygenation rate is the slope of the linear portion of StO_2_ ascent after walking cessation. Time to StO_2_ recovery is the time elapsed to achieve the baseline StO_2_ after walking cessation. Finally, maximum StO_2_ is assessed as the maximum StO_2_ achieved during the recovery period after exercise.

**Figure 2.**
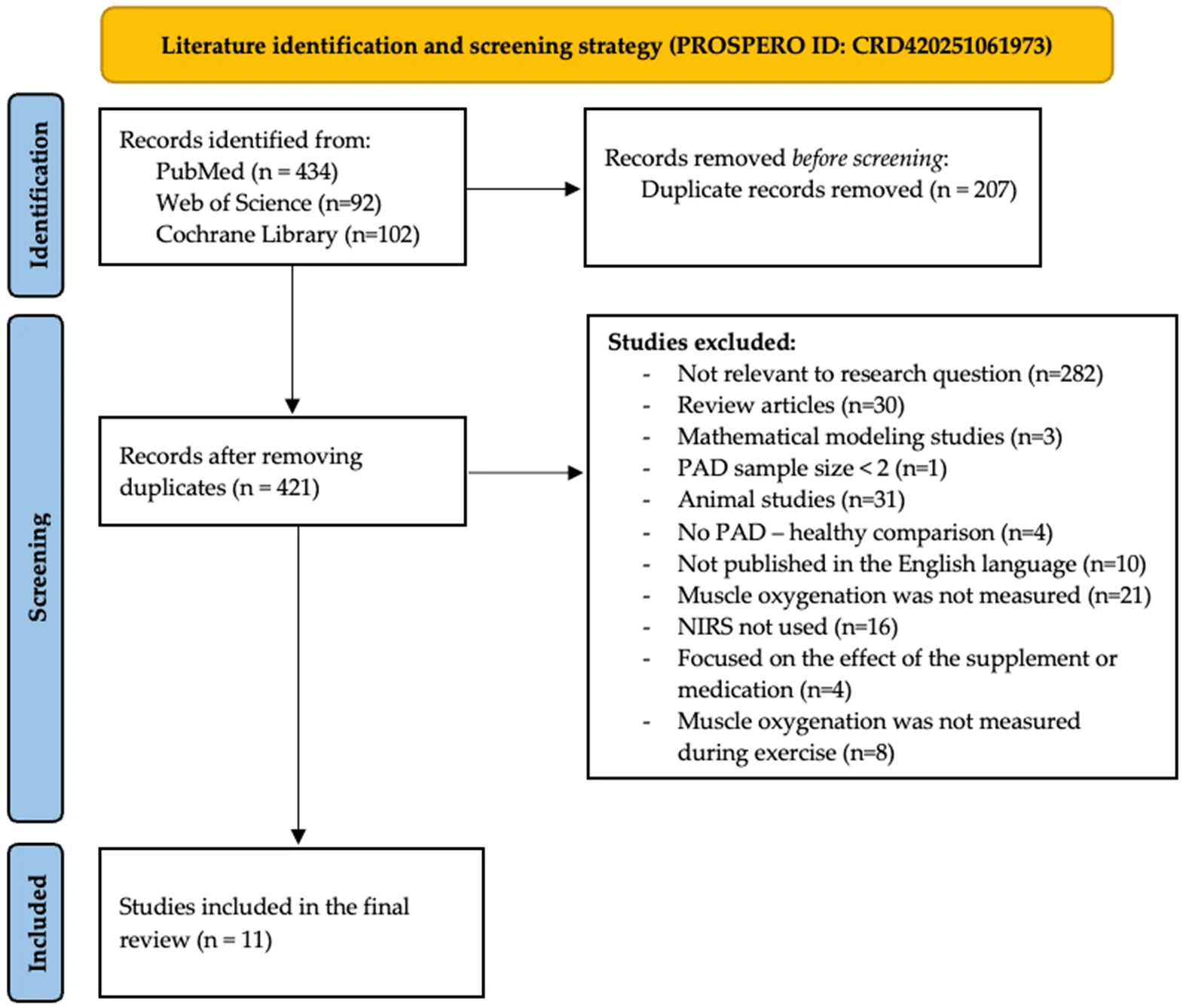
Flowchart of the search and article selection strategy. After searching for keywords in the selected databases, 11 articles remained. No additional articles were added by searching the reference lists.

**Table 1.** Definition of frequent terms used for muscle oxygenation measurements in different studies. Hem: hemoglobin [40]; Hb: hemoglobin [40]; Mb: myoglobin [40]; HbO_2_: oxyhemoglobin [40]; O_2_Hb: oxyhemoglobin [40]; HHb: deoxyhemoglobin [40].

NIRS-Derived Variables	Frequent Terms and Equations
Total hemoglobin	Total [hem]; total [Hb + Mb]; [tHb] [40]
Oxyhemoglobin	Oxy [hem]; oxy [Hb + Mb]; O_2_Hb; HbO_2_ [40]
Deoxyhemoglobin	Deoxy [hem]; deoxy [Hb + Mb]; HHb [40]
Muscle Oxygenation	Tissue oxygen saturation (StO_2_) [43]; StO_2_% = (O_2_Hb/O_2_Hb + HHb) × 100 Muscle oxygen saturation (SmO_2_) [44]; SmO_2_% = (O_2_Hb/O_2_Hb + HHb) × 100 Tissue oxygenation index (TOI) [41]; TOI% = (O_2_Hb/O_2_Hb + Hb) × 100 Tissue saturation index (TSI) [42,45]; TSI% = (O_2_Hb/O_2_Hb + HHb) × 100

**Table 2.** PICO terms used to find articles for this systematic review.

Acronym	Definition	Description
P	Population	Patients with PAD.
I	Intervention	Exercise (typically this means an acute exercise session).
C	Comparison	Baseline (resting) versus exercise and post-exercise (recovery) values, and healthy versus PAD.
O	Outcomes	NIRS muscle oxygenation measurements.

**Table 3.** Overview of the methodological quality of articles based on the Critical Review Form: Quantitative Studies. This form assesses the key methodological components, such as the purpose of the study, method, statistical analysis, results, and conclusion. N/A: Not applicable. 1: Was the purpose stated clearly? 2: Literature: Was the relevant and background literature reviewed? 3: Was the sample described in detail? 4: Was the sample size justified? 5: Was the statistical significance reported for the results? 6: Were the analysis method(s) appropriate? 7: Were clinical characteristics reported? 8: Were dropouts reported? 9: Were the conclusions appropriate based on the study results?

Authors	1	2	3	4	5	6	7	8	9
Song-Young Park, et al. 2022 [9].	Yes	Yes	Yes	N/A	Yes	Yes	Yes	N/A	Yes
Matthew Fuglestad, et al. 2020 [32].	Yes	Yes	Yes	N/A	Yes	Yes	Yes	Yes	Yes
J. Carter Luck, et al. 2017 [79].	Yes	Yes	Yes	Yes	Yes	Yes	Yes	N/A	Yes
F Manfredini, et al., 2017 [80].	Yes	Yes	Yes	Yes	Yes	Yes	Yes	N/A	Yes
F Manfredini, et al. 2009 [46].	Yes	Yes	Yes	N/A	Yes	Yes	Yes	N/A	Yes
DT Ubbink, et al. 2006 [81].	Yes	Yes	Yes	N/A	Yes	Yes	Yes	Yes	Yes
Anthony J. Comerota, et al. 2003 [77].	Yes	Yes	Yes	N/A	Yes	Yes	Yes	N/A	Yes
Anselm Egun, et al. 2002 [76].	Yes	Yes	Yes	N/A	Yes	Yes	Yes	Yes	Yes
G.J. Kemp, et al. 2001 [82].	Yes	Yes	Yes	N/A	Yes	Yes	Yes	Yes	Yes
H. Miriam Kooijman, et al. 1997 [78].	Yes	Yes	Yes	N/A	Yes	Yes	Yes	N/A	Yes
Kevin K. McCully, et al. 1994 [83].	Yes	Yes	Yes	N/A	Yes	Yes	Yes	Yes	Yes

**Table 4.** Reference table. Characteristics (summary and details) of the articles included. NIRS: near-infrared spectroscopy. ABI: ankle-brachial index. ICT: initial claudication time. ACT: absolute claudication time. Gardner treadmill test: Patients perform eight 2 min walking sessions for a maximum of 16 min at a constant 2 mph speed. The incline started at 0% and increased by 2% every 2 min until it reached 14% [77]. O_2_Hb: oxyhemoglobin [40,76]. Oxygen index: difference between oxygenated and deoxygenated hemoglobin [76]. HHb: deoxyhemoglobin [34,76]. tHb: total hemoglobin. Hb: hemoglobin.

Article	Sample (n)	Study	Study Objective	Outcome Measurements	Results
Song-Young Park, et al. 2022 [9].	10 patients with claudication pain and 11 healthy subjects	Observational study	(1) Determine the effect of chronic ischemia on skeletal microcirculatory function. (2) Determine the correlation between leg ischemia and muscle mitochondrial function and StO_2_.	An NIRS device was attached to the calf muscle to measure StO_2_ at baseline and during walking and recovery using the Gardner-Skinner protocol.	Patients with PAD and healthy subjects showed no significant difference between baseline StO_2_ prior to exercise and maximum StO_2_ after exercise. However, patients with PAD showed greater StO_2_ reduction during walking and slower StO_2_ recovery compared with healthy subjects. Minimum StO_2_ during walking did not differ between patients with PAD and healthy subjects.
Matthew Fuglestad, et al. 2020 [32].	40 patients with PAD and 10 control subjects	Observational study	(1) Examine NIRS profile differences in patients with PAD vs. healthy subjects. (2) Determine how walking limitations relate to NIRS parameters during exercise training.	Calf muscle StO_2_ was measured for 3 min at baseline/rest, during the graded treadmill test, and 30 min after the test. Healthy control subjects performed a 540 s treadmill test. Additionally, all subjects performed computerized angiography and a 6 min walking test.	Patients with PAD and healthy subjects showed similar baseline StO_2_ levels. However, patients with PAD showed faster StO_2_ drop, greater StO_2_ fluctuation, greater decrease in StO_2_ during, and longer recovery after treadmill walking compared with healthy subjects. In patients with PAD, a strong correlation between calf muscle hypoxia and walking capacity during the 6 min walking test was observed.
J. Carter Luck, et al. 2017 [79]	Experiment 1: 8 patients with PAD and 8 healthy subjects. Experiment 2: 7 patients with PAD and 7 healthy subjects.	Controlled experimental study	Compare blood pressure (BP) and StO_2_ responses to different exercise intensities during plantar flexion exercise in patients with PAD vs. healthy subjects.	NIRS assessed calf muscle StO_2_ in the most symptomatic leg of patients with PAD. Experiment 1: After a 3 min baseline, subjects performed supine single-leg contraction (most symptomatic leg) at 30 contractions/min with progressive weights (0.5–7kg) for up to 14 min, stopping if fatigue or pain exceed 5/10. Experiment 2: The same exercise was performed but at 20 contraction/min, short pauses during exercise, and constant weights for 14 min.	Experiment 1: Patients with PAD showed significantly greater StO_2_ reduction during exercise compared with healthy subjects. At the time of fatigue, patients with PAD showed threefold greater StO_2_ reduction compared with healthy subjects. Experiment 2: Due to the lower StO_2_ level at baseline in patients with PAD compared with healthy subjects, StO_2_ was measured as the percentage relative changes with respect to the baseline at 1 min and 14 min. No significant changes were observed at 1 min, while a significant change was observed at 14 min during exercise.
F. Manfredini, et al. 2017 [80]	80 patients with PAD and 13 healthy subjects	Observational study	Assess NIRS feasibility, validity, and diagnostic accuracy during dynamic ambulatory test to diagnose foot perfusion in PAD.	Subjects performed 10 toe flexion repetitions with NIRS attached to the dorsum of their feet. Healthy subjects repeated the test with increased tight blood flow restriction. ABI and the area under the oxygenated hemoglobin curve were measured. Reliability was assessed by repeating the measurement in two trials, and validity was evaluated by finding the association among toe flex curve area, oxygen delivery, ABI, and ankle pressure.	Higher levels of blood flow restriction in healthy subjects resulted in larger O_2_Hb deficit during toe flexion, as shown by a larger area under the curve (more negative). In patients with PAD, the most symptomatic legs showed significantly greater area under curve of O_2_Hb compared with healthy legs, with a weak correlation with dorsal pedis artery pressure. Toe flexion is an effective test to evaluate foot perfusion and PAD even in the presence or absence of ABI measurements.
F Manfredini, et al. 2009 [46]	67 patients with PAD and 28 healthy subjects	Observational study	Assess if NIRS can determine calf muscle deoxygenation during treadmill walking in patients with PAD.	NIRS was attached to the calf muscle to measure the variation in oxygenation, deoxygenation, tHB and differential oxygenation. The test included 1 min warm-up, followed by treadmill walking at 1.5 km/h, increasing the speed every 10 min by 0.1 km/h. The test was stopped when the patients were unable to continue due to fatigue, dyspnea, or claudication.	The areas under curves for Hb, deoxygenation, and differential oxygenation were significantly differed in symptomatic and non-symptomatic legs. However, no significant differences in tHb were observed between symptomatic and non-symptomatic legs. Patients with PAD showed higher compensatory heart rate during exercise compared with healthy subjects. NIRS measurements were used to effectively quantify muscle metabolic response in PAD.
DT Ubbink, et al. 2006 [81]	45 patients with different stages of leg ischemia and 20 healthy subjects	Prospective comparative study	Determine the reproducibility and clinical applicability of NIRS in patients with leg ischemia.	Reproductivity of various diagnostic tests, leg perfusion, and blood pressure were measured at rest, during exercise, and after changing posture. NIRS was attached to the calf muscle’s lateral side. NIRS was used to measure StO_2_ for 5 min while subjects walked on a treadmill at 3.1km/h speed and 8% incline.	NIRS could not detect resting StO_2_ differences across various PAD stages or correlate with ABI. However, lower ABI after exercise was correlated with the StO_2_ reduction measured by NIRS. Although ABI was able to detect leg ischemia at rest, NIRS could not detect it due to its inability to differentiate between the presence or absence of vascular disease. NIRS is a reliable measurement due to its ability to detect greater Hb desaturation during exercise in patients with PAD compared with controls. NIRS outcomes did not correlate with macro- and microcirculatory measurements.
Anthony J. Comerota, et al. 2003 [77]	14 patients with PAD and 35 healthy subjects	Prospective cross-sectional study	(1) Identify calf StO_2_ and ABI correlation in patients with PAD. (2) Identify the sensitivity of muscle oxygenation to identify PAD. (3) Identify the relationship between StO_2_ and claudication symptoms. (4) Assess the safety of NIRS in screening for PAD.	Patients rested before the progressive treadmill test. The ABI was measured before and at 5 min intervals following the test. NIRS was attached to the calf muscle to record StO_2_ 5 min before, during, and 20 min after the test. Outcomes included baseline StO_2_, peak exercise StO_2_, absolute percentage changes between baseline and peak exercise, StO_2_ values at ICT and ACT, and StO_2_ at 50% (T50) and 100% (T100) after stopping the exercise.	ABI was significantly different between PAD and healthy subjects, while no significant differences in StO_2_ were observed. Patients with PAD showed significantly lower StO_2_ at peak exercise, greater absolute difference between baseline and peak exercise, greater percentage changes from baseline to peak exercise, and longer recovery time at (T50) and (T100) compared with healthy subjects. A significant correlation was observed between T50 and ABI.
Anselm Egun, et al. 2002 [76]	16 patients with PAD, and 7 age-matched and 7 young healthy control subjects.	Comparative study	Determine exercise-induced calf muscle ischemia in patients with PAD using NIRS.	NIRS equipment was attached to the patients’ most affected calf and the right calf of the healthy subjects. StO_2_ was recorded 10 min before, during, and 10 min after treadmill walking. Subjects first walked for 1 min at 3.2 kph and 10° incline. After a 20 min rest, the maximum walking capacity was assessed at the same speed and incline. In this test, patients with PAD walked until pain forced them to stop, while healthy subjects walked for 7 min.	During treadmill walking, all subjects showed O_2_Hb reduction. Patients showed greater HHb during exercise compared with healthy age-matched and young subjects; the differences were not significant. O_2_Hb and tHb were lower during the maximum-exercise test in patients with PAD vs. healthy subjects. In recovery, patients showed significantly increased O_2_Hb, tHb, and oxygen index, while healthy subjects showed no significant changes. ABI at rest was correlated with HHb but not with tHb or the oxygenated index during a maximum-walking test.
G.J. Kemp, et al. 2001 [82]	11 patients with PAD and 9 healthy subjects	Case–control study	Measure calf muscle oxygenation and adenosine triphosphate (ATP) in patients with PAD by using magnetic resonance spectroscopy (MRS) and NIRS.	NIRS and MRS measured calf muscle ATP level and StO_2_ during isometric plantar flexion at 50% and 75% of maximum voluntary contraction for 2–4 min, following 5 min of recovery. Each subject repeated the plantar flexion test three times, and the results were averaged.	Compared with healthy subjects, patients with PAD showed faster and greater deoxygenation during exercise and slower reoxygenation after exercise.
H. Miriam Kooijman, et al. 1997 [78]	11 patients with PAD and 15 healthy subjects	Experimental study	Evaluate the utility of NIRS to assess muscle oxygenation hemodynamics in patients with PAD compared with healthy subjects.	NIRS equipment was attached to the left calf of healthy subjects and the patients’ most symptomatic calf muscle. Part 1: Venous occlusion was performed at rest using a cuff, followed by at least 30 min of rest. Part 2: Subjects performed standard treadmill walking for 4 min at 3.2 km/h speed and 6° incline. Subjects reported ICT and walked until pain forced them to stop. Part 3: The same walking protocol in part 1 was repeated to measure StO_2_ during and after treadmill walking.	Patients with PAD showed significantly greater deoxygenation during walking compared to healthy subjects. Following the walking exercise, no significant difference was observed in blood flow between patients with PAD and healthy subjects. Patients with PAD showed significantly slower resaturation rates, longer recovery time measured by NIRS, and longer recovery time measured by ABI compared with healthy subjects.
Kevin K. McCully, et al. 1994 [83]	8 patients with PAD, 20 healthy older adult, and 6 healthy young subjects.	Comparative study	(1) Compare post-reoxygenation rate in PAD vs. healthy older adults. (2) Compare post-reoxygenation rate in healthy older vs. young adults. (3) Compare calf StO_2_ depletion in PAD vs. healthy subjects.	ABI was measured before exercise. NIRS equipment was attached to the lateral soleus while subjects performed one-leg plantar flexion exercise every 5 s for one minute. Recovery time and StO_2_ were recorded for 3–6 min after exercise (one leg in healthy subjects and both legs in patients with PAD). StO_2_ was measured for 5 min in the standing position before and during the progressive treadmill test. During the treadmill test, ICT and ACT were recorded.	There was a significant difference in the rate of muscle oxygen saturation after plantar flexion exercise between the most symptomatic and less symptomatic legs of patients with PAD. Compared with healthy young and old subjects, patients with PAD showed greater and faster deoxygenation during walking and longer recovery after the walking test. The recovery time after exercise for the most symptomatic leg was longer compared with the less symptomatic leg.

## Data Availability

The data presented in this study are available on request from the corresponding author. The data are not publicly available due to privacy and personal data protection, as well as the need for context to properly interpret.

## Abbreviations

The following abbreviations are used in this manuscript:

PAD: Peripheral artery disease
StO_2_: Tissue oxygen saturation
SmO_2_: Muscle oxygen saturation
TOI: Tissue oxygenation index
Mb: Myoglobin
tHb: Total hemoglobin
O_2_Hb: Oxyhemoglobin
Hb: Hemoglobin
HHb: Deoxyhemoglobin

## References

[1] Lakatta EG. Age-Associated Cardiovascular Changes in Health: Impact on Cardiovascular Disease in Older Persons. Heart Fail. Rev. 2002, 7, 29–49. 10.1023/a:1013797722156 11790921

[2] Strait JB; Lakatta EG. Aging-Associated Cardiovascular Changes and Their Relationship to Heart Failure. Heart Fail. Clin. 2012, 8, 143. 10.1016/j.hfc.2011.08.011 PMC3223374 22108734

[3] Fallahtafti F; Salamifar Z; Hassan M; Rahman H; Pipinos I; Myers SA. Joint Angle Variability Is Altered in Patients with Peripheral Artery Disease after Six Months of Exercise Intervention. Entropy 2022, 24, 1422. 10.3390/e24101422 PMC9602135 37420442

[4] Fallahtafti F; Samson K; Salamifar SZ; Johanning J; Pipinos I; Myers SA. Three Months of Wearing an Ankle Foot Orthosis Improves the Spatiotemporal Gait Characteristics in Patients with Peripheral Artery Disease. J. Appl. Biomech. 2025, 41, 200–206. 10.1123/jab.2024-0083 40043720

[5] Ismaeel A; Miserlis D; Papoutsi E; Haynatzki G; Bohannon WT; Smith RS; Eidson JL; Casale GP; Pipinos II; Koutakis P. Endothelial Cell-Derived pro-Fibrotic Factors Increase TGF-B1 Expression by Smooth Muscle Cells in Response to Cycles of Hypoxia-Hyperoxia. Biochim. Biophys. Acta Mol. Basis Dis. 2022, 1868, 166278. 10.1016/j.bbadis.2021.166278 PMC8629962 34601016

[6] Mietus CJ; Lackner TJ; Karvelis PS; Willcockson GT; Shields CM; Lambert NG; Koutakis P; Fuglestad MA; Hernandez H; Haynatzki GR; Abnormal Microvascular Architecture, Fibrosis, and Pericyte Characteristics in the Calf Muscle of Peripheral Artery Disease Patients with Claudication and Critical Limb Ischemia. J. Clin. Med. 2020, 9, 2575. 10.3390/jcm9082575 PMC7464726 32784470

[7] Duscha BD; Kraus WE; Jones WS; Robbins JL; Piner LW; Huffman KM; Allen JD; Annex BH. Skeletal Muscle Capillary Density Is Related to Anaerobic Threshold and Claudication in Peripheral Artery Disease. Vasc. Med. 2020, 25, 411–418. 10.1177/1358863X20945794 PMC8297535 32841100

[8] Baum O; Djonov V; Ganster M; Widmer M; Baumgartner I. Arteriolization of Capillaries and FGF-2 Upregulation in Skeletal Muscles of Patients with Chronic Peripheral Arterial Disease. Microcirculation 2005, 12, 527–537. 10.1080/10739680591003413 16147469

[9] Park SY; Pekas EJ; Anderson CP; Kambis TN; Mishra PK; Schieber MN; Wooden TSK; Thompson JR; Kim KS; Pipinos II. Impaired Microcirculatory Function, Mitochondrial Respiration, and Oxygen Utilization in Skeletal Muscle of Claudicating Patients with Peripheral Artery Disease. Am. J. Physiol. Heart Circ. Physiol. 2022, 322, H867–H879. 10.1152/ajpheart.00690.2021 PMC9018007 35333113

[10] Gardner AW; Montgomery PS; Wang M; Shen B. Association between Calf Muscle Oxygen Saturation with Ambulatory Function and Quality of Life in Symptomatic Patients with Peripheral Artery Disease. J. Vasc. Surg. 2020, 72, 632–642. 10.1016/j.jvs.2019.09.057 PMC7387166 32081480

[11] McDermott MMG. Lower Extremity Manifestations of Peripheral Artery Disease: The Pathophysiologic and Functional Implications of Leg Ischemia. Circ. Res. 2015, 116, 1540–1550. 10.1161/CIRCRESAHA.114.303517 PMC4410164 25908727

[12] Hiatt WR; Armstrong EJ; Larson CJ; Brass EP. Pathogenesis of the Limb Manifestations and Exercise Limitations in Peripheral Artery Disease. Circ. Res. 2015, 116, 1527–1539. 10.1161/CIRCRESAHA.116.303566 25908726

[13] Shahbad R; Pipinos M; Jadidi M; Desyatova A; Gamache J; MacTaggart J; Kamenskiy A. Structural and Mechanical Properties of Human Superficial Femoral and Popliteal Arteries. Ann. Biomed. Eng. 2024, 52, 794–815. 10.1007/s10439-023-03435-3 PMC11455778 38321357

[14] McDermott MM; Ferrucci L; Gonzalez-Freire M; Kosmac K; Leeuwenburgh C; Peterson CA; Saini S; Sufit R. Skeletal Muscle Pathology in Peripheral Artery Disease: A Brief Review. Arterioscler. Thromb. Vasc. Biol. 2020, 40, 2577–2585. 10.1161/ATVBAHA.120.313831 PMC9571495 32938218

[15] Murrow JR; Brizendine JT; Djire B; Young HJ; Rathbun S; Nilsson KR; McCully KK. Near Infrared Spectroscopy-Guided Exercise Training for Claudication in Peripheral Arterial Disease. Eur. J. Prev. Cardiol. 2019, 26, 471–480. 10.1177/2047487318795192 30152245

[16] Reiner Ž; De Sutter J; Ryden L; Mirrakhimov E; Pogosova N; Dolzhenko M; Fras Z; Kotseva K; Wood D; De Bacquer D. Peripheral Arterial Disease and Intermittent Claudication in Coronary Heart Disease Patients. Int. J. Cardiol. 2021, 322, 227–232. 10.1016/j.ijcard.2020.09.004 32918939

[17] Matsui S; Tamura N; Hirakawa T; Kobayashi S; Takekoshi N; Murakami E. Assessment of Working Skeletal Muscle Oxygenation in Patients with Chronic Heart Failure. Am. Heart J. 1995, 129, 690–695. 10.1016/0002-8703(95)90317-8 7900619

[18] Jones B; Hesford CM; Cooper CE. The Use of Portable NIRS to Measure Muscle Oxygenation and Haemodynamics during a Repeated Sprint Running Test. Adv. Exp. Med. Biol. 2013, 789, 185–191. 10.1007/978-1-4614-7411-1_26 23852494

[19] Nygren A; Rennerfelt K; Zhang Q. Detection of Changes in Muscle Oxygen Saturation in the Human Leg: A Comparison of Two near-Infrared Spectroscopy Devices. J. Clin. Monit. Comput. 2014, 28, 57–62. 10.1007/s10877-013-9494-x 23846128

[20] Bauer TA; Brass EP; Hiatt WR. Impaired Muscle Oxygen Use at Onset of Exercise in Peripheral Arterial Disease. J. Vasc. Surg. 2004, 40, 488–493. 10.1016/j.jvs.2004.06.025 15337878

[21] Nagasawa T. Slower Recovery Rate of Muscle Oxygenation after Sprint Exercise in Long-Distance Runners Compared with That in Sprinters and Healthy Controls. J. Strength Cond. Res. 2013, 27, 3360–3366. 10.1519/JSC.0b013e3182908fcc 23604001

[22] Fu TC; Wang CH; Lin PS; Hsu CC; Cherng WJ; Huang SC; Liu MH; Chiang CL; Wang JS. Aerobic Interval Training Improves Oxygen Uptake Efficiency by Enhancing Cerebral and Muscular Hemodynamics in Patients with Heart Failure. Int. J. Cardiol. 2013, 167, 41–50. 10.1016/j.ijcard.2011.11.086 22197120

[23] Lanzi S; Pousaz A; Fresa M; Besson C; Desgraz B; Gremeaux-Bader V; Mazzolai L. Short-Duration Aerobic High-Intensity Intervals versus Moderate Exercise Training Intensity in Patients with Peripheral Artery Disease: Study Protocol for a Randomised Controlled Trial (the Angiof-HIIT Study). BMJ Open 2024, 14, e081883. 10.1136/bmjopen-2023-081883 PMC11029310 38631833

[24] McCully K; Posner J. Measuring Exercise-Induced Adaptations and Injury with Magnetic Resonance Spectroscopy. Int. J. Sports Med. 1992, 13, S147–S149. 10.1055/s-2007-1024621 1483756

[25] Komiyama T; Shigematsu H; Yasuhara H; Muto T. Near-Infrared Spectroscopy Grades the Severity of Intermittent Claudication in Diabetics More Accurately than Ankle Pressure Measurement. Br. J. Surg. 2000, 87, 459–466. 10.1046/j.1365-2168.2000.01381.x 10759743

[26] Yamamoto K; Miyata T; Onozuka A; Koyama H; Ohtsu H; Nagawa H. Plantar Flexion as an Alternative to Treadmill Exercise for Evaluating Patients with Intermittent Claudication. Eur. J. Vasc. Endovasc. Surg. 2007, 33, 325–329. 10.1016/j.ejvs.2006.10.012 17137808

[27] Mohler ER; Lech G; Supple GE; Wang H; Chance B. Impaired Exercise-Induced Blood Volume in Type 2 Diabetes with or without Peripheral Arterial Disease Measured by Continuous-Wave near-Infrared Spectroscopy. Diabetes Care 2006, 29, 1856–1859. 10.2337/dc06-0182 16873792

[28] Komiyama T; Shigematsu H; Yasuhara H; Muto T. An Objective Assessment of Intermittent Claudication by Near-Infrared Spectroscopy. Eur. J. Vasc. Surg. 1994, 8, 294–296. 10.1016/s0950-821x(05)80144-6 8013679

[29] Murakami M; Katsumura T; Hamaoka T; Osada T; Sako T; Higuchi H; Esaki K; Kime R; Shimomitsu T. Effects of Epinephrine and Lactate on the Increase in Oxygen Consumption of Nonexercising Skeletal Muscle after Aerobic Exercise. J. Biomed. Opt. 2000, 5, 406. 10.1117/1.1289143 11092428

[30] Hamaoka T; McCully KK; Quaresima V; Yamamoto K; Chance B. Near-Infrared Spectroscopy/Imaging for Monitoring Muscle Oxygenation and Oxidative Metabolism in Healthy and Diseased Humans. J. Biomed. Opt. 2007, 12, 062105. 10.1117/1.2805437 18163808

[31] Sanni AA; McCully KK. Interpretation of Near-Infrared Spectroscopy (NIRS) Signals in Skeletal Muscle. J. Funct. Morphol. Kinesiol. 2019, 4, 28. 10.3390/jfmk4020028 PMC7739319 33467344

[32] Fuglestad MA; Hernandez H; Gao Y; Ybay H; Schieber MN; Brunette KE; Myers SA; Casale GP; Pipinos II. A Low-Cost, Wireless near-Infrared Spectroscopy Device Detects the Presence of Lower Extremity Atherosclerosis as Measured by Computed Tomographic Angiography and Characterizes Walking Impairment in Peripheral Artery Disease. J. Vasc. Surg. 2020, 71, 946–957. 10.1016/j.jvs.2019.04.493 PMC7501596 31445826

[33] Kawaguchi K; Hayashi Y; Sekikawa K; Tabusadani M; Inamizu T; Onari K; Bhambhani Y. Vastus Lateralis Oxygenation during Prolonged Cycling in Healthy Males. Appl. Physiol. Nutr. Metab. 2006, 31, 48–55. 10.1139/h05-001 16604141

[34] Feldmann AM; Erlacher D; Pfister S; Lehmann R. Muscle Oxygen Dynamics in Elite Climbers during Finger-Hang Tests at Varying Intensities. Sci. Rep. 2020, 10, 3040. 10.1038/s41598-020-60029-y PMC7033122 32080325

[35] Shapiro NI; Arnold R; Sherwin R; O’Connor J; Najarro G; Singh S; Lundy D; Nelson T; Trzeciak SW; Jones AE. The Association of Near-Infrared Spectroscopy-Derived Tissue Oxygenation Measurements with Sepsis Syndromes, Organ Dysfunction and Mortality in Emergency Department Patients with Sepsis. Crit. Care 2011, 15, R223. 10.1186/cc10463 PMC3334769 21939529

[36] Varis E; Pettilä V; Wilkman E. Near-Infrared Spectroscopy in Adult Circulatory Shock: A Systematic Review. J. Intensiv. Care Med. 2020, 35, 943–962. 10.1177/0885066620907307 32077780

[37] Hampton DA; Schreiber MA. Near Infrared Spectroscopy: Clinical and Research Uses. Transfusion 2013, 53, 52S–58S. 10.1111/trf.12036 23301973

[38] Coelho R; Tavares J; Marinheiro C; Costa C; Ferreira S; Gregório T. The Effectiveness of NIRS Technology to the Early Diagnosis of Lower Limb Ischemia in Patients on Peripheral VA ECMO: A Systematic Review and Meta-Analysis. Intensiv. Crit. Care Nurs. 2025, 89, 104039. 10.1016/j.iccn.2025.104039 40233544

[39] Gardner AW; Montgomery PS; Wang M; Liang M. Effects of Long-Term Home Exercise in Participants with Peripheral Artery Disease. J. Am. Heart Assoc. Cardiovasc. Cerebrovasc. Dis. 2023, 12, 29755. 10.1161/JAHA.122.029755 PMC10727372 37929770

[40] Barstow TJ. Understanding near Infrared Spectroscopy and Its Application to Skeletal Muscle Research. J. Appl. Physiol. 2019, 126, 1360–1376. 10.1152/japplphysiol.00166.2018 30844336

[41] Al-Rawi PG; Smielewski P; Kirkpatrick PJ. Evaluation of a Near-Infrared Spectrometer (NIRO 300) for the Detection of Intracranial Oxygenation Changes in the Adult Head. Stroke 2001, 32, 2492–2499. 10.1161/hs1101.098356 11692006

[42] McManus CJ; Collison J; Cooper CE. Performance Comparison of the MOXY and PortaMon Near-Infrared Spectroscopy Muscle Oximeters at Rest and during Exercise. J. Biomed. Opt. 2018, 23, 015007. 10.1117/1.JBO.23.1.015007 29368457

[43] El Masry M; Li R; Balasubramani GK; Roy S; Sen CK; Gnyawali SC. Comparative Assessment of Healthy Tissue Oxygenation Using Near-Infrared Imaging, Transcutaneous Oxygen Measurement, and Plethysmography. Sci. Rep. 2025, 15, 30424. 10.1038/s41598-025-15767-2 PMC12365204 40830190

[44] Feldmann A; Schmitz R; Erlacher D. Near-Infrared Spectroscopy-Derived Muscle Oxygen Saturation on a 0% to 100% Scale: Reliability and Validity of the Moxy Monitor. J. Biomed. Opt. 2019, 24, 115001. 10.1117/1.JBO.24.11.115001 PMC7003144 31741352

[45] Page MJ; McKenzie JE; Bossuyt PM; Boutron I; Hoffmann TC; Mulrow CD; Shamseer L; Tetzlaff JM; Akl EA; Brennan SE; The PRISMA 2020 Statement: An Updated Guideline for Reporting Systematic Reviews. BMJ 2021, 372, n71. 10.1136/bmj.n71 PMC8005924 33782057

[46] Manfredini F; Malagoni AM; Felisatti M; Mandini S; Mascoli F; Manfredini R; Basaglia N; Zamboni P. A Dynamic Objective Evaluation of Peripheral Arterial Disease by Near-Infrared Spectroscopy. Eur. J. Vasc. Endovasc. Surg. 2009, 38, 441–448. 10.1016/j.ejvs.2009.06.011 19625198

[47] Bae SY; Hamaoka T; Katsumura T; Shiga T; Ohno H; Haga S. Comparison of Muscle Oxygen Consumption Measured by near Infrared Continuous Wave Spectroscopy during Supramaximal and Intermittent Pedalling Exercise. Int. J. Sports Med. 2000, 21, 168–174. 10.1055/s-2000-8880 10834347

[48] Bhambhani Y; Maikala R; Esmail S. Oxygenation Trends in Vastus Lateralis Muscle during Incremental and Intense Anaerobic Cycle Exercise in Young Men and Women. Eur. J. Appl. Physiol. 2001, 84, 547–556. 10.1007/s004210000372 11482550

[49] Azuma K; Homma S; Kagaya A. Oxygen Supply-Consumption Balance in the Thigh Muscles during Exhausting Knee-Extension Exercise. J. Biomed. Opt. 2000, 5, 97. 10.1117/1.429974 10938772

[50] Kemp GJ; Hands LJ; Ramaswami G; Taylor DJ; Nicolaides A; Amato A; Radda GK. Calf Muscle Mitochondrial and Glycogenolytic ATP Synthesis in Patients with Claudication Due to Peripheral Vascular Disease Analysed Using 31P Magnetic Resonance Spectroscopy. Clin. Sci. 1995, 89, 581–590. 10.1042/cs0890581 8549076

[51] Takaishi T; Ishida K; Katayama K; Yamazaki K; Yamamoto T; Moritani T. Effect of Cycling Experience and Pedal Cadence on the Near-Infrared Spectroscopy Parameters. Med. Sci. Sports Exerc. 2002, 34, 2062–2071. 10.1097/00005768-200212000-00030 12471317

[52] Neary JP; McKenzie DC; Bhambhani YN. Effects of Short-Term Endurance Training on Muscle Deoxygenation Trends Using NIRS. Med. Sci. Sports Exerc. 2002, 34, 1725–1732. 10.1097/00005768-200211000-00006 12439075

[53] Marwood S; Bowtell JL. Effects of Glutamine and Hyperoxia on Pulmonary Oxygen Uptake and Muscle Deoxygenation Kinetics. Eur. J. Appl. Physiol. 2007, 99, 149–161. 10.1007/s00421-006-0324-4 17115180

[54] Ding H; Wang G; Lei W; Wang R; Huang L; Xia Q; Wu J. Non-Invasive Quantitative Assessment of Oxidative Metabolism in Quadriceps Muscles by near Infrared Spectroscopy. Br. J. Sports Med. 2001, 35, 441. 10.1136/bjsm.35.6.441 PMC1724436 11726485

[55] Legrand R; Ahmaidi S; Moalla W; Chocquet D; Marles A; Prieur F; Mucci P. O2 Arterial Desaturation in Endurance Athletes Increases Muscle Deoxygenation. Med. Sci. Sports Exerc. 2005, 37, 782–788. 10.1249/01.mss.0000161806.47058.40 15870632

[56] Watanabe T; Matsushita M; Nishikimi N; Sakurai T; Komori K; Nimura Y. Near-Infrared Spectroscopy with Treadmill Exercise to Assess Lower Limb Ischemia in Patients with Atherosclerotic Occlusive Disease. Surg. Today 2004, 34, 849–854. 10.1007/s00595-004-2833-2 15449155

[57] Watanabe S; Ishii C; Takeyasu N; Ajisaka R; Nishina H; Morimoto T; Sakamoto K; Eda K; Ishiyama M; Saito T; Assessing Muscle Vasodilation Using Near-Infrared Spectroscopy in Cardiac Patients. Circ. J. 2005, 69, 802–814. 10.1253/circj.69.802 15988106

[58] Kankaanpää M; Colier WN; Taimela S; Anders C; Airaksinen O; Kokko-Aro SM; Hänninen O. Back Extensor Muscle Oxygenation and Fatigability in Healthy Subjects and Low Back Pain Patients during Dynamic Back Extension Exertion. Pathophysiology 2005, 12, 267–273. 10.1016/j.pathophys.2005.09.013 16289763

[59] Kell RT; Bhambhani Y. Relationship between Erector Spinae Static Endurance and Muscle Oxygenation-Blood Volume Changes in Healthy and Low Back Pain Subjects. Eur. J. Appl. Physiol. 2006, 96, 241–248. 10.1007/s00421-005-0072-x 16261387

[60] Maikala RV; Bhambhani YN. In Vivo Lumbar Erector Spinae Oxygenation and Blood Volume Measurements in Healthy Men during Seated Whole-Body Vibration. Exp. Physiol. 2006, 91, 853–866. 10.1113/expphysiol.2006.033696 16740640

[61] Yoshitake Y; Ue H; Miyazaki M; Moritani T. Assessment of Lower-Back Muscle Fatigue Using Electromyography, Mechanomyography, and near-Infrared Spectroscopy. Eur. J. Appl. Physiol. 2001, 84, 174–179. 10.1007/s004210170001 11320632

[62] Inuzuka K; Unno N; Mitsuoka H; Yamamoto N; Ishimaru K; Sagara D; Suzuki M; Konno H. Intraoperative Monitoring of Penile and Buttock Blood Flow during Endovascular Abdominal Aortic Aneurysm Repair. Eur. J. Vasc. Endovasc. Surg. 2006, 31, 359–365. 10.1016/j.ejvs.2005.09.019 16364666

[63] McKeon MD; Albert WJ; Neary JP. Assessment of Neuromuscular and Haemodynamic Activity in Individuals with and without Chronic Low Back Pain. Dyn. Med. 2006, 5, 6. 10.1186/1476-5918-5-6 PMC1489922 16734915

[64] Legrand R; Marles A; Prieur F; Lazzari S; Blondel N; Mucci P. Related Trends in Locomotor and Respiratory Muscle Oxygenation during Exercise. Med. Sci. Sports Exerc. 2007, 39, 91–100. 10.1249/01.mss.0000241638.90348.67 17218889

[65] Esaki K; Hamaoka T; Rådegran G; Boushel R; Hansen J; Katsumura T; Haga S; Mizuno M. Association between Regional Quadriceps Oxygenation and Blood Oxygen Saturation during Normoxic One-Legged Dynamic Knee Extension. Eur. J. Appl. Physiol. 2005, 95, 361–370. 10.1007/s00421-005-0008-5 16096839

[66] Tamaki T; Uchiyama S; Tamura T; Nakano S. Changes in Muscle Oxygenation during Weight-Lifting Exercise. Eur. J. Appl. Physiol. Occup. Physiol. 1994, 68, 465–469. 10.1007/BF00599514 7957136

[67] de Oliveira GV; Volino-Souza M; Barros-Santos E; Conte-Junior CA; Alvares TS. The Influence of Cardiovascular Risk Factors on Near-Infrared Spectroscopy-Derived Muscle Oxygen Saturation during Exercise Recovery in Older Adults. Sport Sci. Health 2022, 18, 1221–1228.

[68] Ichimura S; Murase N; Osada T; Kime R; Homma T; Ueda C; Nagasawa T; Motobe M; Hamaoka T; Katsumura T. Age and Activity Status Affect Muscle Reoxygenation Time after Maximal Cycling Exercise. Med. Sci. Sports Exerc. 2006, 38, 1277–1281. 10.1249/01.mss.0000227312.08599.f1 16826024

[69] Kutsuzawa T; Shioya S; Kurita D; Haida M; Yamabayashi H. Effects of Age on Muscle Energy Metabolism and Oxygenation in the Forearm Muscles. Med. Sci. Sports Exerc. 2001, 33, 901–906. 10.1097/00005768-200106000-00008 11404654

[70] Figoni SF; Kunkel CF; Scremin AME; Asher A; Banks NL; Rivera A; Tin JK; Cohen B. Effects of Exercise Training on Calf Tissue Oxygenation in Men with Intermittent Claudication. PM&R 2009, 1, 932–940. 10.1016/j.pmrj.2009.08.453 19854422

[71] Almushayt SJ; Hussain S; Wilkinson DJ; Selby NM. A Systematic Review of the Acute Effects of Hemodialysis on Skeletal Muscle Perfusion, Metabolism, and Function. Kidney Int. Rep. 2020, 5, 307–317. 10.1016/j.ekir.2019.12.012 PMC7056853 32154452

[72] Akobeng AK. Principles of Evidence Based Medicine. Arch. Dis. Child. 2005, 90, 837–840. 10.1136/adc.2005.071761 PMC1720507 16040884

[73] Lockwood C; Porritt K; Munn Z; Rittenmeyer L; Salmond S; Bjerrum M; Loveday H; Carrier J; Stannard D. Chapter 2: Systematic Reviews of Qualitative Evidence. JBI Rev. Man. 2019.

[74] Swinnen E; Kerckhofs E. Compliance of Patients Wearing an Orthotic Device or Orthopedic Shoes: A Systematic Review. J. Bodyw. Mov. Ther. 2015, 19, 759–770. 10.1016/j.jbmt.2015.06.008 26592234

[75] Barclay L; McDonald R; Lentin P. Social and Community Participation Following Spinal Cord Injury: A Critical Review. Int. J. Rehabil. Res. 2015, 38, 1–19. 10.1097/MRR.0000000000000085 25305008

[76] Egun A; Farooq V; Torella F; Cowley R; Thorniley MS; McCollum CN. The Severity of Muscle Ischemia during Intermittent Claudication. J. Vasc. Surg. 2002, 36, 89–93. 10.1067/mva.2002.123678 12096263

[77] Comerota AJ; Throm RC; Kelly P; Jaff M. Tissue (Muscle) Oxygen Saturation (StO_2_): A New Measure of Symptomatic Lower-Extremity Arterial Disease. J. Vasc. Surg. 2003, 38, 724–729. 10.1016/s0741-5214(03)01032-2 14560221

[78] Kooijman HM; Hopman MTE; Colier WNJM; Van Der Vliet JA; Oeseburg B. Near Infrared Spectroscopy for Noninvasive Assessment of Claudication. J. Surg. Res. 1997, 72, 1–7. 10.1006/jsre.1997.5164 9344707

[79] Luck JC; Miller AJ; Aziz F; Radtka JF; Proctor DN; Leuenberger UA; Sinoway LI; Muller XMD. Blood Pressure and Calf Muscle Oxygen Extraction during Plantar Flexion Exercise in Peripheral Artery Disease. J. Appl. Physiol. 2017, 123, 2–10. 10.1152/japplphysiol.01110.2016 PMC5538812 28385920

[80] Manfredini F; Lamberti N; Rossi T; Mascoli F; Basaglia N; Zamboni P. A Toe Flexion NIRS Assisted Test for Rapid Assessment of Foot Perfusion in Peripheral Arterial Disease: Feasibility, Validity, and Diagnostic Accuracy. Eur. J. Vasc. Endovasc. Surg. 2017, 54, 187–194. 10.1016/j.ejvs.2017.04.013 28571673

[81] Ubbink DT; Koopman B. Near-Infrared Spectroscopy in the Routine Diagnostic Work-up of Patients with Leg Ischaemia. Eur. J. Vasc. Endovasc. Surg. 2006, 31, 394–400. 10.1016/j.ejvs.2005.10.025 16359878

[82] Kemp GJ; Roberts N; Bimson WE; Bakran A; Harris PL; Gilling-Smith GL; Brennan J; Rankin A; Frostick SP. Mitochondrial Function and Oxygen Supply in Normal and in Chronically Ischemic Muscle: A Combined 31P Magnetic Resonance Spectroscopy and near Infrared Spectroscopy Study in Vivo. J. Vasc. Surg. 2001, 34, 1103–1110. 10.1067/mva.2001.117152 11743568

[83] McCully KK; Halber C; Posner JD. Exercise-Induced Changes in Oxygen Saturation in the Calf Muscles of Elderly Subjects with Peripheral Vascular Disease. J. Gerontol. 1994, 49, B128–B134. 10.1093/geronj/49.3.b128 8169330

[84] Koutakis P; Miserlis D; Myers SA; Kim JKS; Zhu Z; Papoutsi E; Swanson SA; Haynatzki G; Ha DM; Carpenter LA; Abnormal Accumulation of Desmin in Gastrocnemius Myofibers of Patients with Peripheral Artery Disease: Associations with Altered Myofiber Morphology and Density, Mitochondrial Dysfunction and Impaired Limb Function. J. Histochem. Cytochem. 2015, 63, 256–269. 10.1369/0022155415569348 PMC4374059 25575565

[85] Ha DM; Carpenter LC; Koutakis P; Swanson SA; Zhu Z; Hanna M; DeSpiegelaere HK; Pipinos II; Casale GP. Transforming Growth Factor-Beta 1 Produced by Vascular Smooth Muscle Cells Predicts Fibrosis in the Gastrocnemius of Patients with Peripheral Artery Disease. J. Transl. Med. 2016, 14, 39. 10.1186/s12967-016-0790-3 PMC4743093 26847457

[86] Zahner GJ; Spaulding KA; Ramirez JL; Schaller MS; Walker SC; Hills NK; Gasper WJ; Grenon SM. Characterizing the Relationship between Flow-Mediated Vasodilation and Radial Artery Tonometry in Peripheral Artery Disease. J. Surg. Res. 2018, 224, 121–131. 10.1016/j.jss.2017.11.062 PMC5844355 29506827

[87] Makris KI; Nella AA; Zhu Z; Swanson SA; Casale GP; Gutti TL; Judge AR; Pipinos II. Mitochondriopathy of Peripheral Arterial Disease. Vascular 2007, 15, 336–343. 10.2310/6670.2007.00054 18053417

[88] Pipinos II; Judge AR; Zhu Z; Selsby JT; Swanson SA; Johanning JM; Baxter BT; Lynch TG; Dodd SL. Mitochondrial Defects and Oxidative Damage in Patients with Peripheral Arterial Disease. Free Radic. Biol. Med. 2006, 41, 262–269. 10.1016/j.freeradbiomed.2006.04.003 16814106

[89] Pipinos II; Judge AR; Selsby JT; Zhu Z; Swanson SA; Nella AA; Dodd SL. The Myopathy of Peripheral Arterial Occlusive Disease: Part 1. Functional and Histomorphological Changes and Evidence for Mitochondrial Dysfunction. Vasc. Endovasc. Surg. 2007, 41, 481–489. 10.1177/1538574407311106 18166628

[90] Pipinos II; Judge AR; Selsby JT; Zhu Z; Swanson SA; Nella AA; Dodd SL. The Myopathy of Peripheral Arterial Occlusive Disease: Part 2. Oxidative Stress, Neuropathy, and Shift in Muscle Fiber Type. Vasc. Endovasc. Surg. 2008, 42, 101–112. 10.1177/1538574408315995 PMC12282609 18390972

[91] Allen MF; Park SY; Kwak Y. Oxidative Stress and Vascular Dysfunction: Potential Therapeutic Targets and Therapies in Peripheral Artery Disease. Microvasc. Res. 2024, 155, 104713. 10.1016/j.mvr.2024.104713 38914307

[92] Kim K; Anderson EM; Scali ST; Ryan TE. Skeletal Muscle Mitochondrial Dysfunction and Oxidative Stress in Peripheral Arterial Disease: A Unifying Mechanism and Therapeutic Target. Antioxidants 2020, 9, 1304. 10.3390/antiox9121304 PMC7766400 33353218

[93] Gratl A; Wipper S; Frese JP; Raude B; Greiner A; Pesta D. The Role of Mitochondrial Function in Peripheral Arterial Disease: Insights from Translational Studies. Int. J. Mol. Sci. 2021, 22, 8478. 10.3390/ijms22168478 PMC8395190 34445191

[94] Signorelli SS; Marino E; Scuto S; Di Raimondo D. Pathophysiology of Peripheral Arterial Disease (PAD): A Review on Oxidative Disorders. Int. J. Mol. Sci. 2020, 21, 4393. 10.3390/ijms21124393 PMC7352779 32575692

[95] Brass EP; Hiatt WR; Green S. Skeletal Muscle Metabolic Changes in Peripheral Arterial Disease Contribute to Exercise Intolerance: A Point-Counterpoint Discussion. Vasc. Med. 2004, 9, 293–301. 10.1191/1358863x04vm572ra 15678622

[96] Gardner AW; Montgomery PS; Wang M; Shen B; Afaq A; Khurana A. Light and Moderate Intensity Physical Activity Are Associated with Better Ambulation, Quality of Life, and Vascular Measurements in Patients with Claudication. J. Vasc. Surg. 2022, 75, 1739–1749. 10.1016/j.jvs.2021.12.071 PMC9038648 34999217

[97] Yu G; Durduran T; Lech G; Zhou C; Chance B; Mohler ER; Yodh AG. Time-Dependent Blood Flow and Oxygenation in Human Skeletal Muscles Measured with Noninvasive near-Infrared Diffuse Optical Spectroscopies. J. Biomed. Opt. 2005, 10, 024027. 10.1117/1.1884603 15910100

[98] Yasunobu Y; Oudiz RJ; Sun XG; Hansen JE; Wasserman K. End-Tidal Pco_2_ Abnormality and Exercise Limitation in Patients with Primary Pulmonary Hypertension. Chest 2005, 127, 1637–1646. 10.1378/chest.127.5.1637 15888840

[99] Hart CR; Layec G; Trinity JD; Le Fur Y; Gifford JR; Clifton HL; Richardson RS. Oxygen Availability and Skeletal Muscle Oxidative Capacity in Patients with Peripheral Artery Disease: Implications from In Vivo and In Vitro Assessments. Am. J. Physiol. Heart Circ. Physiol. 2018, 315, H897–H909. 10.1152/ajpheart.00641.2017 PMC6230899 29932772

[100] Baker WB; Li Z; Schenkel SS; Chandra M; Busch DR; Englund EK; Schmitz KH; Yodh AG; Floyd TF; Mohler ER. Effects of Exercise Training on Calf Muscle Oxygen Extraction and Blood Flow in Patients with Peripheral Artery Disease. J. Appl. Physiol. 2017, 123, 1599–1609. 10.1152/japplphysiol.00585.2017 PMC5814687 28982943

[101] Paulino Geisel P; Pantuso Monteiro D; de Oliveira Nascimento I; Gomes Pereira DA. Evaluation of Functional Capacity and Muscle Metabolism in Individuals with Peripheral Arterial Disease with and without Diabetes. J. Vasc. Surg. 2022, 75, 671–679. 10.1016/j.jvs.2021.08.082 34921971

[102] Cornelis N; Chatzinikolaou PN; Buys R; De Wilde C; Fourneau I; Claes J; Goetschalckx K; Cornelissen V. Near Infrared Spectroscopy to Evaluate the Effect of a Hybrid Exercise Programme on Peripheral Muscle Metabolism in Patients with Intermittent Claudication: An Exploratory PROSECO-IC Sub Study. J. Sports Sci. 2022, 40, 1031–1041. 10.1080/02640414.2022.2045062 35271414

[103] Prior SJ; Chrencik MT; Christensen E; Kundi R; Ryan AS; Addison O; Lal BK. An Exercise Stress Test for Contrast-Enhanced Duplex Ultrasound Assessment of Lower Limb Muscle Perfusion in Patients with Peripheral Arterial Disease. J. Vasc. Surg. 2024, 79, 397–404. 10.1016/j.jvs.2023.10.005 PMC10969459 37844848

[104] Davidson BP; Hodovan J; Mason OR; Moccetti F; Gupta A; Muller M; Belcik JT; Annex BH; Lindner JR. Limb Perfusion During Exercise Assessed by Contrast Ultrasound Varies According to Symptom Severity in Patients with Peripheral Artery Disease. J. Am. Soc. Echocardiogr. 2019, 32, 1086–1094.e3. 10.1016/j.echo.2019.05.001 PMC6732010 31235422

[105] Meneses AL; Nam MCY; Bailey TG; Magee R; Golledge J; Hellsten Y; Keske MA; Greaves K; Askew CD. Leg Blood Flow and Skeletal Muscle Microvascular Perfusion Responses to Submaximal Exercise in Peripheral Arterial Disease. Am. J. Physiol. Heart Circ. Physiol. 2018, 315, H1425–H1433. 10.1152/ajpheart.00232.2018 30095999

[106] Davidson BP; Belcik JT; Landry G; Linden J; Lindner JR. Exercise versus Vasodilator Stress Limb Perfusion Imaging for the Assessment of Peripheral Artery Disease. Echocardiography 2017, 34, 1187–1194. 10.1111/echo.13601 PMC5568936 28664576

[107] Bakermans AJ; Wessel CH; Zheng KH; Groot PFC; Stroes ESG; Nederveen AJ. Dynamic Magnetic Resonance Measurements of Calf Muscle Oxygenation and Energy Metabolism in Peripheral Artery Disease. J. Magn. Reson. Imaging 2020, 51, 98–107. 10.1002/jmri.26841 PMC6916546 31218803

[108] Anderson JD; Epstein FH; Meyer CH; Hagspiel KD; Wang H; Berr SS; Harthun NL; Weltman A; DiMaria JM; West AM; Multifactorial Determinants of Functional Capacity in Peripheral Arterial Disease. Uncoupling of Calf Muscle Perfusion and Metabolism. J. Am. Coll. Cardiol. 2009, 54, 628–635. 10.1016/j.jacc.2009.01.080 PMC2768062 19660694

[109] Sporkin HL; Patel TR; Betz Y; Mathew R; Schumann CL; Meyer CH; Kramer CM. Chemical Exchange Saturation Transfer Magnetic Resonance Imaging Identifies Abnormal Calf Muscle-Specific Energetics in Peripheral Artery Disease. Circ. Cardiovasc. Imaging 2022, 15, 511–519. 10.1161/CIRCIMAGING.121.013869 PMC9310050 35861977

[110] Kriel Y; Kwintowski A; Feka K; Windsor M; Young G; Walker M; Askew CD. Near-Infrared Spectroscopy During Reactive Hyperemia for the Assessment of Lower Limb Vascular Function. J. Vis. Exp. 2024, 2024, e66511. 10.3791/66511 38587379

[111] Grassi B; Quaresima V. Near-Infrared Spectroscopy and Skeletal Muscle Oxidative Function in Vivo in Health and Disease: A Review from an Exercise Physiology Perspective. J. Biomed. Opt. 2016, 21, 091313. 10.1117/1.JBO.21.9.091313 27443955

[112] Jones S; Chiesa ST; Chaturvedi N; Hughes AD. Recent Developments in Near-Infrared Spectroscopy (NIRS) for the Assessment of Local Skeletal Muscle Microvascular Function and Capacity to Utilise Oxygen. Artery Res. 2016, 16, 25. 10.1016/j.artres.2016.09.001 PMC5134760 27942271

[113] Moudgil S; Kalra N; Prabhakar N; Dhiman RK; Behera A; Chawla YK; Khandelwal N. Comparison of Contrast Enhanced Ultrasound with Contrast Enhanced Computed Tomography for the Diagnosis of Hepatocellular Carcinoma. J. Clin. Exp. Hepatol. 2017, 7, 222. 10.1016/j.jceh.2017.03.003 PMC5620356 28970709

[114] Bajwa A; Wesolowski R; Patel A; Saha P; Ludwinski F; Smith A; Nagel E; Modarai B. Assessment of Tissue Perfusion in the Lower Limb Current Methods and Techniques under Development. Circ. Cardiovasc. Imaging 2014, 7, 836–843. 10.1161/CIRCIMAGING.114.002123 25227236

[115] Jones EJ; Atherton PJ; Piasecki M; Phillips BE. Contrast-Enhanced Ultrasound Repeatability for the Measurement of Skeletal Muscle Microvascular Blood Flow. Exp. Physiol. 2023, 108, 549–553. 10.1113/EP091034 PMC10103852 36738267

[116] Galanakis N; Maris TG; Kontopodis N; Tsetis K; Kehagias E; Tsetis D. Perfusion Imaging Techniques in Lower Extremity Peripheral Arterial Disease. Br. J. Radiol. 2022, 95, 20211203. 10.1259/bjr.20211203 PMC10996332 35522774

[117] Elsaid NMH; Peters DC; Galiana G; Sinusas AJ. Clinical Physiology: The Crucial Role of MRI in Evaluation of Peripheral Artery Disease. Am. J. Physiol. Circ. Physiol. 2024, 326, H1304–H1323. 10.1152/ajpheart.00533.2023 PMC11381027 38517227

[118] Ghadimi M; Thomas A. Magnetic Resonance Imaging Contraindications. In StatPearls [Internet]; StatPearls Publishing: Tampa/St. Petersburg, FL, USA, 2025. 31869133

[119] Tafti D; Singh V; Cheung ME; Firstenberg MS. Duplex Ultrasound. In Epidemiology of Peripheral Vascular Disease; Springer: Berlin/Heidelberg, Germany, 2025; pp. 41–54.

[120] Cosgrove D; Lassau N. Imaging of Perfusion Using Ultrasound. Eur. J. Nucl. Med. Mol. Imaging 2010, 37, 65–85. 10.1007/s00259-010-1537-7 20640418

[121] Nguyen T; Davidson BP. Contrast Enhanced Ultrasound Perfusion Imaging in Skeletal Muscle. J. Cardiovasc. Imaging 2019, 27, 163. 10.4250/jcvi.2019.27.e31 PMC6669180 31161755

[122] Tuesta M; Yáñez-Sepúlveda R; Verdugo-Marchese H; Mateluna C; Alvear-Ordenes I. Near-Infrared Spectroscopy Used to Assess Physiological Muscle Adaptations in Exercise Clinical Trials: A Systematic Review. Biology 2022, 11, 1073. 10.3390/biology11071073 PMC9312707 36101451

[123] Miranda-Fuentes C; Chirosa-Ríos LJ; Guisado-Requena IM; Delgado-Floody P; Jerez-Mayorga D. Changes in Muscle Oxygen Saturation Measured Using Wireless Near-Infrared Spectroscopy in Resistance Training: A Systematic Review. Int. J. Environ. Res. Public Health 2021, 18, 4293. 10.3390/ijerph18084293 PMC8074101 33919532

[124] Hoffman JR; Im J; Rundell KW; Kang J; Nioka S; Speiring BA; Kime R; Chance B. Effect of Muscle Oxygenation during Resistance Exercise on Anabolic Hormone Response. Med. Sci. Sports Exerc. 2003, 35, 1929–1934. 10.1249/01.MSS.0000093613.30362.DF 14600561

[125] da Fonseca Cerqueira MMB; Bastos NSSG; da Silva DAR; Gregori D; Magalhães LBNC; Pimentel MMW. Accuracy of Ankle-Brachial Index in Screening for Peripheral Arterial Disease in People with Diabetes. PLoS ONE 2024, 19, e0309083. 10.1371/journal.pone.0309083 PMC11501029 39446913

[126] Maruhashi T; Matsui S; Yusoff FM; Kishimoto S; Kajikawa M; Higashi Y. Falsely Normalized Ankle-Brachial Index despite the Presence of Lower-Extremity Peripheral Artery Disease: Two Case Reports. J. Med. Case Rep. 2021, 15, 622. 10.1186/s13256-021-03155-z PMC8679997 34920746

[127] Abouhamda A; Alturkstani M; Jan Y. Lower Sensitivity of Ankle-Brachial Index Measurements among People Suffering with Diabetes-Associated Vascular Disorders: A Systematic Review. SAGE Open Med. 2019, 7, 2050312119835038. 10.1177/2050312119835038 PMC6399753 30854203

[128] Van Beekvelt MCP; Borghuis MS; Van Engelen BG; Wevers RA; Colier WN. Adipose Tissue Thickness Affects In Vivo Quantitative Near-IR Spectroscopy in Human Skeletal Muscle. Clin. Sci. 2001, 101, 21–28. 10.1042/cs20000247 11410110

[129] Bopp CM; Townsend DK; Barstow TJ. Characterizing Near-Infrared Spectroscopy Responses to Forearm Post-Occlusive Reactive Hyperemia in Healthy Subjects. Eur. J. Appl. Physiol. 2011, 111, 2753–2761. 10.1007/s00421-011-1898-z 21409404

[130] Craig JC; Broxterman RM; Wilcox SL; Chen C; Barstow TJ. Effect of Adipose Tissue Thickness, Muscle Site, and Sex on near-Infrared Spectroscopy Derived Total-[Hemoglobin + Myoglobin]. J. Appl. Physiol. 2017, 123, 1571–1578. 10.1152/japplphysiol.00207.2017 28935822

